# Developing a digital psychosocial support program for men with low-risk prostate cancer during active surveillance

**DOI:** 10.1016/j.invent.2025.100853

**Published:** 2025-06-25

**Authors:** Kim Donachie, Michel Hansma, Marian Adriaansen, Erik Cornel, Esther Bakker, Lilian Lechner

**Affiliations:** a HAN University of Applied Sciences, Health Academy, Kapittelweg 33, 6525EN Nijmegen, the Netherlands; b Andros Clinics, Meester E.N. van Kleffensstraat 5, 6842 CV Arnhem, the Netherlands; c Open University, Faculty of Psychology, Valkenburgerweg 177, 6419 AT Heerlen, the Netherlands

**Keywords:** Prostate cancer, Active surveillance, Design thinking, Psychosocial support, *E*-health interventions

## Abstract

**Background:**

Active surveillance (AS) is a preferred treatment for men with low- to intermediate-risk prostate cancer, but its psychosocial impact presents challenges. This study used design thinking to develop a digital psychosocial support program aimed at improving quality of life and health outcomes for men on AS.

**Methods:**

The design process followed five phases: Empathy, Define, Ideate, Prototype, and Test. Stakeholder interviews were conducted to generate a problem statement. Brainstorming in the ideation phase conceptualized a self-management application and a framework of the application's features was developed. A prototype was developed in close collaboration with end-users and experts. The testing phase included heuristic evaluations and feedback from patients and healthcare providers.

**Results:**

Interviews during the empathy phase highlighted the need for personalized care, timely information, and holistic and tailored support. The defined problem statement aimed at reducing the psychosocial burden and improving coping mechanisms during the first year of AS. Ideation involved multidisciplinary brainstorming sessions, resulting in the concept of a self-management application with features such as information, appointment preparation, self-reporting of medical results, lifestyle guidance, relaxation exercises, and communication tools. A prototype application was developed. Testing showed strengths in navigation and design, with recommendations for improving error handling and help documentation. Feedback led to refinements enhancing usability and clinical integration.

**Conclusion:**

This study developed a patient-centered self-management application to address psychosocial challenges in AS. By fostering engagement, self-efficacy, and communication, the tool aims to improve outcomes in prostate cancer management. Future clinical studies will evaluate its effectiveness.

## Highlights

• Psychosocial support boosts resilience and wellbeing in prostate cancer patients on active surveillance.
• E-health offers timely, accessible and cost-effective psychosocial support.
• This study used design thinking to create a digital support program for men on active surveillance.
• A user-tested self-management app was developed to ease burdens, boost engagement, and support collaboration.

## Abbreviations

PCa: Prostate Cancer
AS: Active Surveillance
DT: Design Thinking
LR-PCa: Low-Risk Prostate Cancer
PSA: Prostate-Specific Antigen

## 1 Introduction

Cancer care has evolved over recent decades from focusing solely on overall survival to prioritizing the quality of life and positive health outcomes (Haylock et al., 2006). This change necessitates that healthcare providers address the long-term needs of cancer patients, emphasizing patient-centered care that enhances overall well-being while considering cost-effectiveness.

Prostate cancer (PCa) is the second most prevalent malignancy globally and exemplifies the challenges of this transition. In 2020, there were an estimated 1.4 million new PCa cases, making it the most frequently diagnosed cancer in 112 countries (Siegel et al., 2023). With an aging global population, PCa incidence is expected to increase. PCa is categorized into low-risk, intermediate-risk, and high-risk groups, with low-risk prostate cancer (LR-PCa) comprising a third of all new cases (Mottet et al., 2023).

Active surveillance (AS) is the preferred management strategy for LR-PCa. This approach involves regular monitoring of tumor progression through Prostate Specific Antigen (PSA) testing, digital rectal exams, MRI, and periodic biopsies, with curative treatment being initiated only upon tumor progression. AS is safe, cost-effective, and avoids treatment-related complications such as erectile dysfunction and urinary incontinence (Mottet et al., 2023; Parker et al., 2023; Naser-Tavakolian et al., 2023). AS is also becoming a popular option for men with intermediate-risk PCa (de Vos et al., 2023). Despite its benefits, the uncertainty associated with AS can significantly impact the mental health and overall quality of life of men, affecting their adherence to AS protocols and potentially leading to disease progression and poorer health outcomes (Alvisi et al., 2020; Parker et al., 2015; Kinsella et al., 2018).

Research suggests that psychosocial support has been shown to reduce uncertainty, anxiety, and distress among cancer patients (Osborn et al., 2006). However, research on interventions specifically tailored for PCa patients undergoing AS remains limited (Donachie et al., 2022a). Most studies focus on standalone interventions with temporary effects. A comprehensive psychosocial support program integrating various interventions could provide broader and more sustainable benefits, optimizing support during AS and improving adherence (Donachie et al., 2022b).

However, offering such a program to all AS patients could result in unnecessary interventions, higher healthcare costs, and an increased caregiver workload. Digital health technologies may present a viable solution in providing just-in-time, personalized care without raising costs or burdening caregivers (Aapro et al., 2020). These tools are scalable, accessible, and cost-effective (Garg et al., 2018). Therefore, digital health technologies can play an important role in addressing the uncertainty, anxiety, and distress often experienced by AS patients while improving adherence to AS protocols and promoting health outcomes sustainably (Faggini et al., 2019).

Successful implementation of digital psychosocial support within healthcare systems depends on usability, effectiveness, and accessibility (Hasnan et al., 2022). Achieving this requires a user-centered, methodical approach to designing and integrating these technologies (Imison et al., 2016). Therefore, this study aims to systematically develop a digital psychosocial support program for men with PCa by applying design thinking principles to improve their quality of life and health outcomes.

## 2 Methods

### 2.1 Study design

**Fig. 1:**
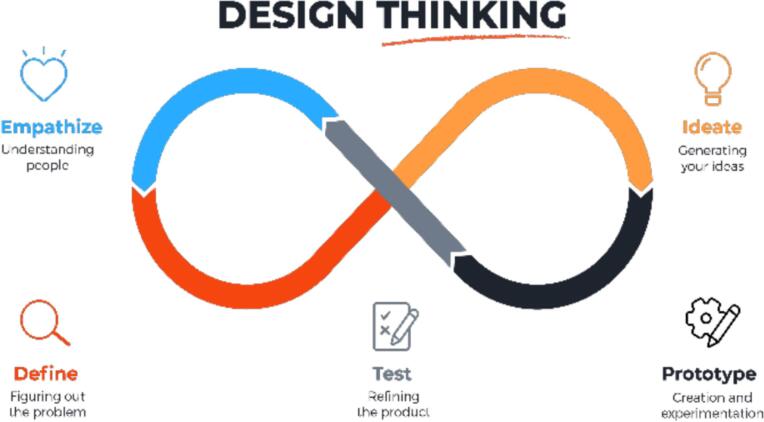
Five phases in design thinking (Oliveira et al., 2020).

A design thinking (DT) approach was used to develop a psychosocial support program for men with PCa during AS. DT is increasingly adopted in healthcare for its human-centered approach to developing patient-centered interventions involving end-users (Oliveira et al., 2020). DT entails five phases in the development process (see Fig. 1). Patient representatives were engaged throughout this study to ensure the program effectively met their needs and preferences. All participants provided verbal informed consent prior to participation, including permission for audio recording (in the empathy interviews) and use of anonymized data for research purposes. The HAN University of Applied Sciences ethical committee provided ethical approval for this study (No. ECO 584.09/24).

The method section provides a concise overview of activities undertaken during the different phases of the Design Thinking (DT) process. The results section offers a detailed account of the activities and outcomes in each phase, highlighting how they informed subsequent phases.

### 2.2 Phase 1. Empathy

A qualitative interview study provided insight into the experiences and needs of men undergoing AS (Donachie et al., 2020). Given that the needs of men undergoing AS were already studied extensively in the beforementioned study by Donachie et al. (2020), additional empathy interviews were conducted during this study and gathered broader insights into the challenges faced by men with PCa during AS as experienced by healthcare professionals. An interview guide was used to execute the interviews (Table 1). The interview guide was developed using the “what, how, why” method, a recommended approach in the empathy phase of design thinking to uncover user needs and experiences (see: Interaction Design Foundation).

**Table 1:** Interview guide.

*1. How do you experience the current healthcare provided to men with prostate cancer during active surveillance?* - What is currently going well? - What is currently not going well? - Can you explain this? *2. When is the care for men with prostate cancer meaningful to you?* - Why? - Can you provide an example? *3. When do you experience the current care to be inadequate?* - Why? - How do you notice this? - Can you provide an example? *4. What would you like to change?* - Why is it important? - How do you envision this? - What is needed for this? - What hindering factors do you foresee? - What would this change yield for both the patient and the healthcare provider?

### 2.3 Phase 2. Define

The insights from the empathy phase were synthesized to develop a problem statement. The findings from the qualitative study and supplementary interviews were deliberated within the research team, leading to the formulation of a clear problem statement.

### 2.4 Phase 3. Ideate

A stakeholder analysis was conducted to identify individuals or groups with a vested interest in or influence on the program. This helped to better understand and incorporate their perspectives, expectations, and concerns. Three brainstorming sessions with key stakeholders were organized, and various brainstorming techniques were used to facilitate creative ideation: the braindump method, the Disney method, mind mapping, and the SCAMPER technique (Serrat, 2017; RGI by Ideanote, 2024). The braindump method enabled participants to quickly capture and share existing thoughts and assumptions, creating a broad starting point for discussion. The Disney method encouraged participants to explore ideas from a more utopian perspective. Mind mapping supported the visual organization and expansion of ideas around central themes and the existing interconnectivity. The SCAMPER technique provided a structured framework to critically review the current care path and identify opportunities to Substitute, Combine, Adapt, Modify, Put to another use, Eliminate, and Reverse aspects of care delivery.

### 2.5 Phase 4. Prototype

Consultations with end-users, including caregivers, patient representatives, information specialists, and regulatory board advisors, helped define the specifications and criteria for the prototype. These criteria included effectiveness, efficiency, inclusivity, accessibility, and user-friendliness. Experts were involved in developing the prototype based on the ideas generated during the ideation phase, which was then tested with a small group of end-users to gather feedback and refine the design.

### 2.6 Phase 5. Test

The refined prototype was tested within a larger group of stakeholders using a heuristic testing approach to assess usability (see Table 2). Eight interviews with end-users and ten interviews with healthcare providers assessed barriers and facilitators for the adoption and implementation of e-health in general, and this e-health tool specifically.

**Table 2:** Heuristic test form.

	0	1	2	3	4	5	Remarks
1. User control and freedom							
Are there clear and easily accessible ways for users to undo or redo actions?							
Can users easily go back to the previous state or page without losing their work or progress?							
2. Consistency and Navigation Clarity							
Is there a consistent layout and design throughout the application?							
Are navigation menus and buttons clearly labeled and logically organized?							
3. Error Prevention							
Are there mechanisms in place to prevent users from making common errors?							
Is there validation for user input (date, PSA value) to ensure it is in the correct format or range?							
4. Ease of Memory Recognition							
Are icons, labels, and symbols used consistently and intuitively throughout the application?							
Is the terminology and language used familiar to the target audience?							
5. Flexibility and Efficiency of Use							
Are there shortcuts or advanced features that experienced users can use for faster interaction?							
Can users customize their settings or interface according to their preferences?							
6. Simplicity and Information Architecture							
Is information presented in a clear and organized manner, without unnecessary complexity?							
Is there a logical hierarchy for content and sections?							
7. Help and Documentation							
Is there accessible documentation or help features for users in need of assistance?							
Are tooltips or contextual help provided for potentially confusing elements?							
8. Progress and Response Notifications							
Do users receive feedback when an action is performed, such as submitting a form or completing a task?							
Are loading indicators or progress bars used when necessary?							
9. Animations and Feedback							
Do animations serve a purpose and provide visual feedback to guide users?							
Do animations enhance the user experience without being distracting?							
10. Affordance for Different States or Properties							
Do interactive elements like buttons and input fields visually indicate their current state (e.g., hover, focus, disabled)?							
Are action-oriented items distinguished from static content?							
11. Cursor Affordance for Actions							
Does the cursor change appropriately to indicate interactive elements or actions (e.g., pointing cursor for clickable items)?							

## 3 Results

### 3.1 Phase 1. Empathy

Participants (N = 5) for the empathy interviews were enrolled using a purposive sampling method and were recruited from the researcher's professional network. To obtain a diverse sample, participants varied in age, gender, region, organization and function (see Table 3). The sample included a patient representative, spouse, nurse practitioner, urologist, and researcher on psychosocial support for men with PCa undergoing AS. A semi-structured interview guide was used to facilitate the interviews (see Table 1). All interviews were conducted using MS Teams and recorded after obtaining verbal consent from the participants. The average duration of the interviews was 33 min (26–40 min).

**Table 3:** Characteristics of participants.

*N* = 5	Function	Gender	Age group	City	Organization	Interview duration (minutes).
Participant 1	*Patient representative*	*M*	*65+*	*The Hague*	*Prostate Cancer Association*	*35:19*
Participant 2	*Spouse*	*F*	*50+*	*Gorinchem*	*Beatrix Hospital*	*26:02*
Participant 3	*Nurse practitioner*	*M*	*30–50*	*Nijmegen*	*CWZ Hospital*	*32:27*
Participant 4	*Urologist*	*M*	*65+*	*Tilburg*	*Andros Clinics*	*40:11*
Participant 5	*Researcher*	*F*	*30–50*	*Rotterdam*	*Erasmus MC*	*33:02*

**Fig. 2:**
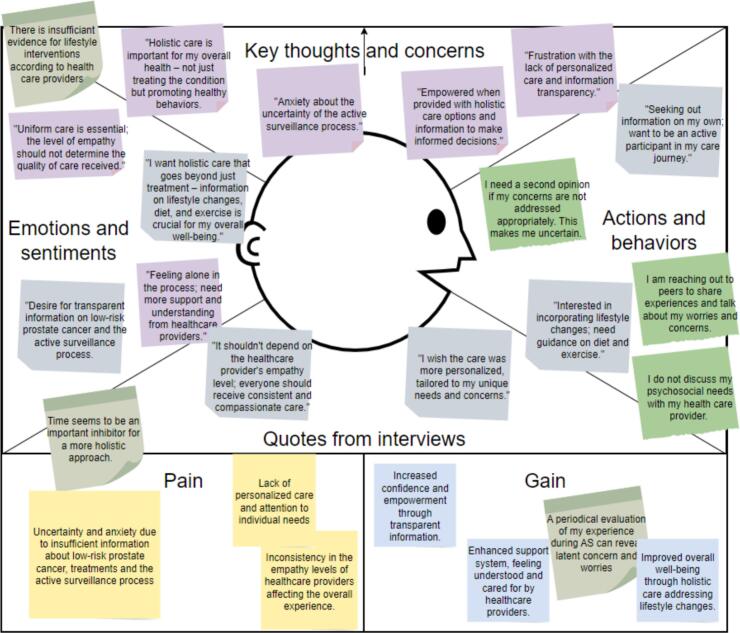
Empathy map.

The recorded interviews were reviewed and analyzed using thematic content analysis. Applying an inductive approach, open coding was used to identify recurring patterns, which were grouped into overarching themes. These themes, along with supporting key quotes, were then translated into the components of an empathy map (Fig. 2), categorizing insights into what users said, did, thought, and felt. Major themes included the need for tailored, personalized care, a more holistic approach with attention to lifestyle, and the importance of just-in-time, reliable information.

### 3.2 Phase 2. Define

The researchers collaboratively defined the problem statement using various tools. The ‘5 WHY’ technique was used to identify the root cause of an issue by repeatedly asking ‘Why?’ until the underlying cause was uncovered (Serrat, 2017). This iterative questioning, typically conducted over five rounds, helps uncover underlying causes and promotes effective solutions. In addition, the collaborative discussions delved into root causes and led to the identification of cohesive themes, offering a comprehensive understanding of challenges faced by men with prostate cancer during active surveillance. This process contributed to the human-centered approach of the study, resulting in the following problem statement:

“*The redesign of support for men with PCa during the first year of the AS, aiming to reduce their psychosocial burden and enhance their ability to cope with the consequences of this trajectory.”*

### 3.3 Phase 3. Ideate

**Fig. 3:**
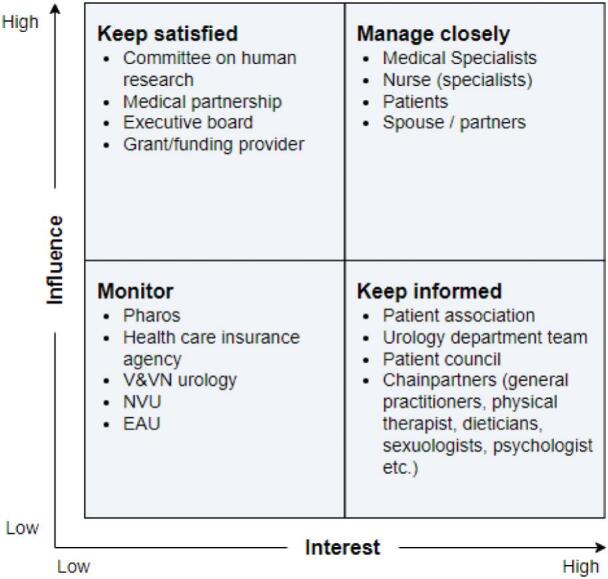
Stakeholder analysis.

A stakeholder analysis identified key individuals involved in the prostate cancer care process (see Fig. 3).

Subsequently, three targeted brainstorming sessions were conducted, involving a diverse panel selected based on the findings of the stakeholder analysis. The panel comprised three patient representatives affiliated with the Prostate Cancer Association, an oncology nurse, two uro-oncological nurse specialists, a student pursuing a Bachelor of Nursing Care currently working as a student nurse in the oncology department, a researcher specializing in media and IT with expertise in inclusivity, and a prostate cancer peer group counselor. The brainstorming sessions, each lasting 90 min, were executed between September 2022 and March 2023, fostering a collaborative environment for creative idea generation. Various brainstorming techniques, such as brain dumping, the Disney-method, the SCAMPER method and mind mapping were used (Serrat, 2017; RGI by Ideanote, 2024). This multi-disciplinary approach harnessed collective expertise and perspectives of the diverse panel and resulted in the conceptualization of a digital health tool as part of the care path for patients undergoing AS: a self-management application.

### 3.4 Phase 4. Prototype

In December 2022, a meeting with three healthcare providers addressed the application's integration into the existing care pathway. The previously conducted Delphi study resulted in a consensus on 13 key interventions to be incorporated into a support program for men on AS. All requirements and conditions for the self-management application were formulated based on the scoping review, Delphi study, and ideation phase (Donachie et al., 2022a; Donachie et al., 2022b). These were subsequently integrated into the prototype program. Experts on data protection, medical device regulation, and inclusivity were consulted. Strict adherence to privacy regulations and data security was stressed. The criteria to avoid classification as a medical device were extensively discussed. To ensure an inclusive application, guidance on accessibility was provided. This resulted in a framework describing the content and features of the prototype self-management application (see Table 4).

**Table 4:** Framework for application content and features.

Buttons	Content	Features
My Information	• Prostate Cancer • Treatment options • Active surveillance • Decision Aids • FAQ	• Accessible and usable • Low health literacy friendly • Link to online tools
My Lifestyle	• Food and Diet • Exercise • Relaxation • FAQ	• Diet plans • Information on food supplements • Reliable references • Advice, schedules, • Exercises
My Appointments	• My protocol • My appointment schedule • Prepare next consultation • Questions for health care provider	• Interactive • Dictaphone function
My Results	• PSA values • Biopsy results • Other medical results • FAQ	• Interactive • Graph and table • Medical Device Regulations complaint
My Contacts	• Contact information health care provider • Peer support contact information	• General Data Protection Act compliant with
My Profile	• General information • Health information	• General Data Protection Act compliant

A researcher and web app developer commenced the development of the prototype in April 2023. The first user test with three healthy male volunteers, all aged 65 and older, was conducted in May 2023. None of the three men had been previously involved in the design and development process. During a one-hour test, the participants were asked to evaluate the application's accessibility, layout, navigational ease and functionality of interactive elements. Feedback was discussed within the research team and incorporated into the ongoing development process.

### 3.5 Phase 5. Test

In September 2023, the prototype was subjected to heuristic testing with a panel of nine experts using three test cases. This panel consisted of two health psychologists, three researchers in e-health applications, two patient representatives, and two nurse practitioners. A simplified heuristic evaluation form was filled out by the participants (see Table 2). The heuristic test displayed that user control and freedom, consistency, navigational ease and recognition were scored positively. Both ‘error prevention’ and ‘help and documentation’ required immediate and further adjustment (see Table 5).

**Table 5:** Results heuristic evaluation.

Domain:	Very poor	Poor	Fair	Neutral	Good	Excellent
1. User control and freedom						
Clear and easy to un/redo actions	0,00 %	0,00 %	16,67 %	33,33 %	33,33 %	16,67 %
Go back without losing work or progress	0,00 %	14,29 %	0,00 %	14,29 %	42,86 %	28,57 %
2. Consistency and Navigation Clarity						
Consistent layout and design	0,00 %	0,00 %	0,00 %	12,50 %	62,50 %	25,00 %
Navigation menus and buttons labeled and organized	0,00 %	0,00 %	0,00 %	12,50 %	25,00 %	62,50 %
3. Error Prevention						
Mechanisms to prevent common errors	16,67 %	0,00 %	0,00 %	16,67 %	50,00 %	16,67 %
Validation for user input to ensure correct format or range	16,67 %	0,00 %	16,67 %	33,33 %	33,33 %	0,00 %
4. Ease of Memory Recognition						
Icons, labels, and symbols used consistently and intuitively	0,00 %	0,00 %	0,00 %	0,00 %	75,00 %	25,00 %
Terminology and language familiar to target audience	0,00 %	0,00 %	0,00 %	28,57 %	28,57 %	42,86 %
5. Flexibility and Efficiency of Use						
Shortcuts or advanced features	0,00 %	0,00 %	14,29 %	57,14 %	14,29 %	14,29 %
Customize settings or interface	14,29 %	14,29 %	0,00 %	57,14 %	14,29 %	0,00 %
6. Simplicity and Information Architecture						
Is information presented clear, organized	0,00 %	0,00 %	14,29 %	14,29 %	28,57 %	42,86 %
Logical hierarchy for content and sections	0,00 %	12,50 %	0,00 %	0,00 %	62,50 %	25,00 %
7. Help and Documentation						
Accessible documentation or help features	0,00 %	33,33 %	50,00 %	0,00 %	0,00 %	16,67 %
Tooltips or contextual help provided	16,67 %	16,67 %	66,67 %	0,00 %	0,00 %	0,00 %
8. Progress and Response Notifications						
Feedback performed actions	0,00 %	14,29 %	28,57 %	28,57 %	14,29 %	14,29 %
Loading indicators or progress bars	28,57 %	0,00 %	14,29 %	28,57 %	0,00 %	28,57 %
9. Animations and Feedback						
Purposeful animations and visual feedback	0,00 %	0,00 %	28,57 %	0,00 %	57,14 %	14,29 %
Animations enhance experience	0,00 %	0,00 %	33,33 %	0,00 %	50,00 %	16,67 %
10. Affordance for Different States or Properties						
Interactive elements visually indicate state (e.g., hover, focus, disabled)	16,67 %	0,00 %	16,67 %	33,33 %	16,67 %	16,67 %
Action-oriented items distinguished from static content	0,00 %	0,00 %	16,67 %	33,33 %	50,00 %	0,00 %
11. Cursor Affordance for Actions						
Cursor changes to indicate interactive elements	0,00 %	16,67 %	0,00 %	33,33 %	16,67 %	33,33 %

In addition, the researchers investigated the barriers and facilitators inhibiting or encouraging the uptake of the application. A panel of eight end-users was interviewed to investigate factors influencing the acceptance and user-friendliness of the application. A panel of ten healthcare providers was interviewed to assess the factors influencing the adoption and implementation in clinical practice. The recommendations emphasized the need for clear visual contrasts in the health application, such as black text on a white background or bold colors like red and blue, with an option for users to customize colors. All options and functions should be arranged on the homepage with icons, accompanied by features like a search function and adjustable font size, to enhance accessibility. Simple, non-medical language is preferred, with explanations provided where necessary. Privacy measures, including a login screen and automatic logout upon app closure, are essential, as well as the exclusion of notifications for advertisements and cookies. Practical guidance for patients on how to use the application, along with training and a test environment for healthcare providers, should be incorporated to ensure effective utilization. Regular updates are necessary to keep the app accurate and reliable.

**Fig. 4:**
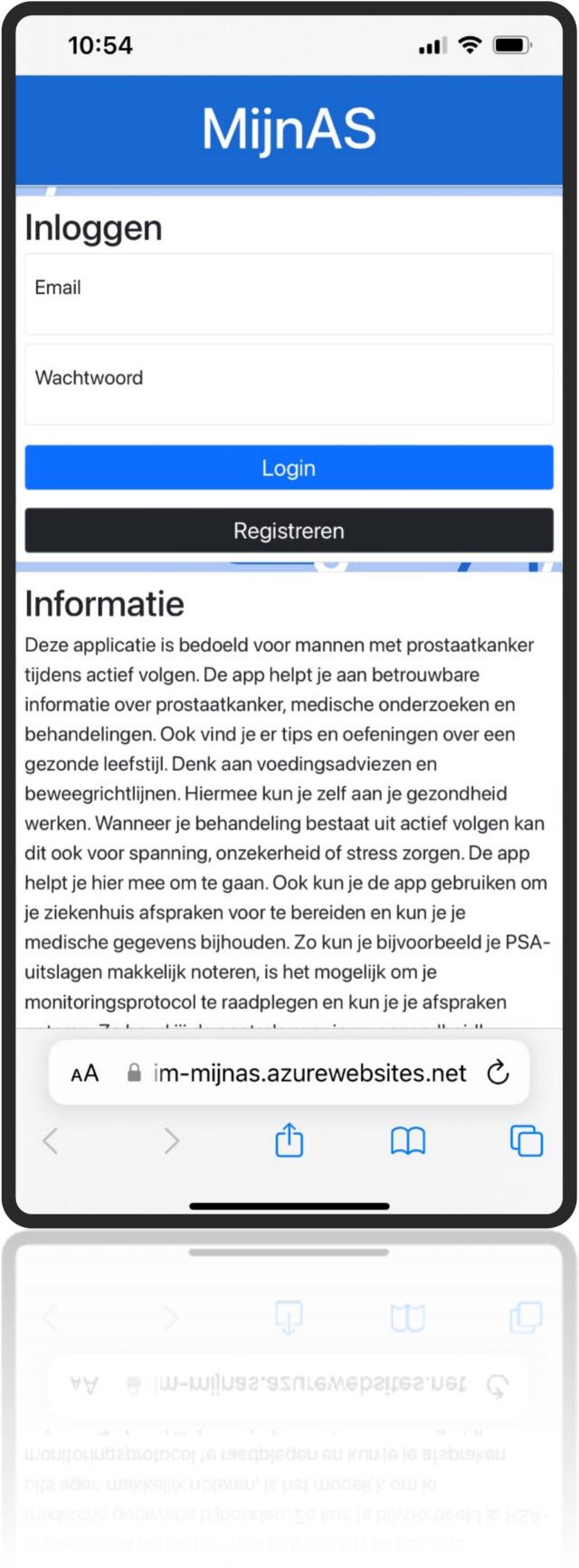
Self-management application login screen.

**Fig. 5:**
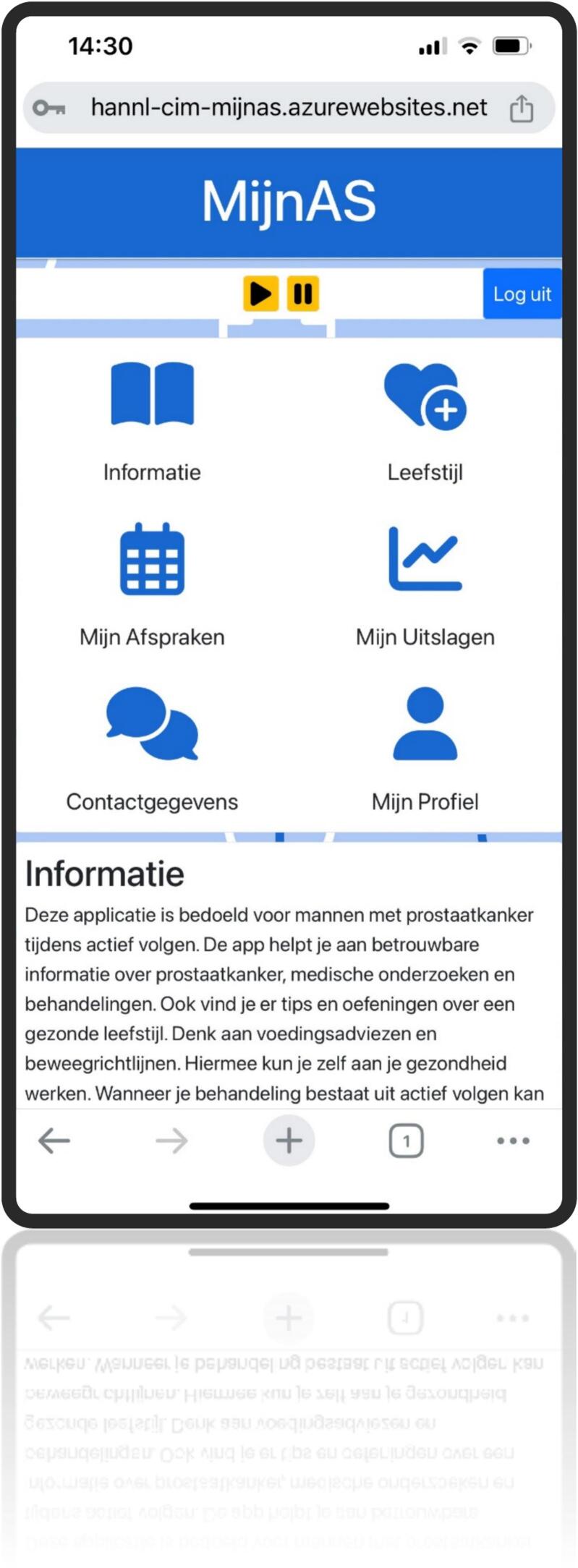
Self management application Home Screen.

**Fig. 6:**
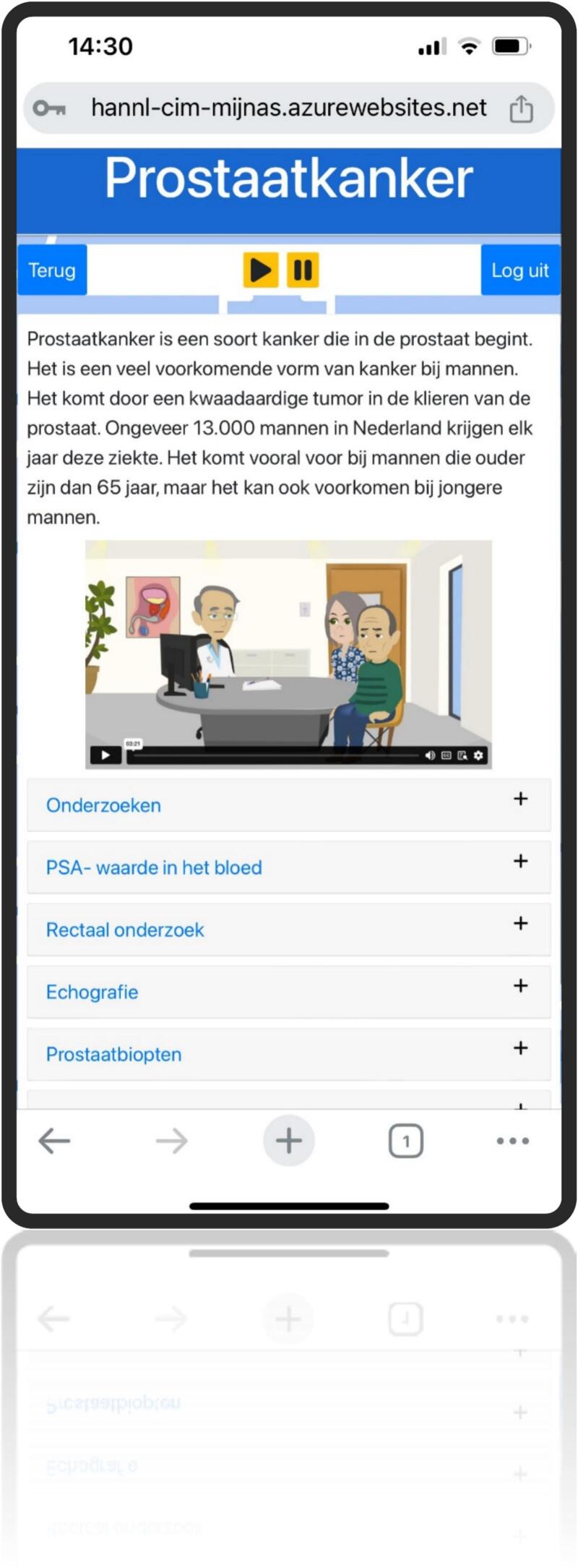
Self management application Information.

**Fig. 7:**
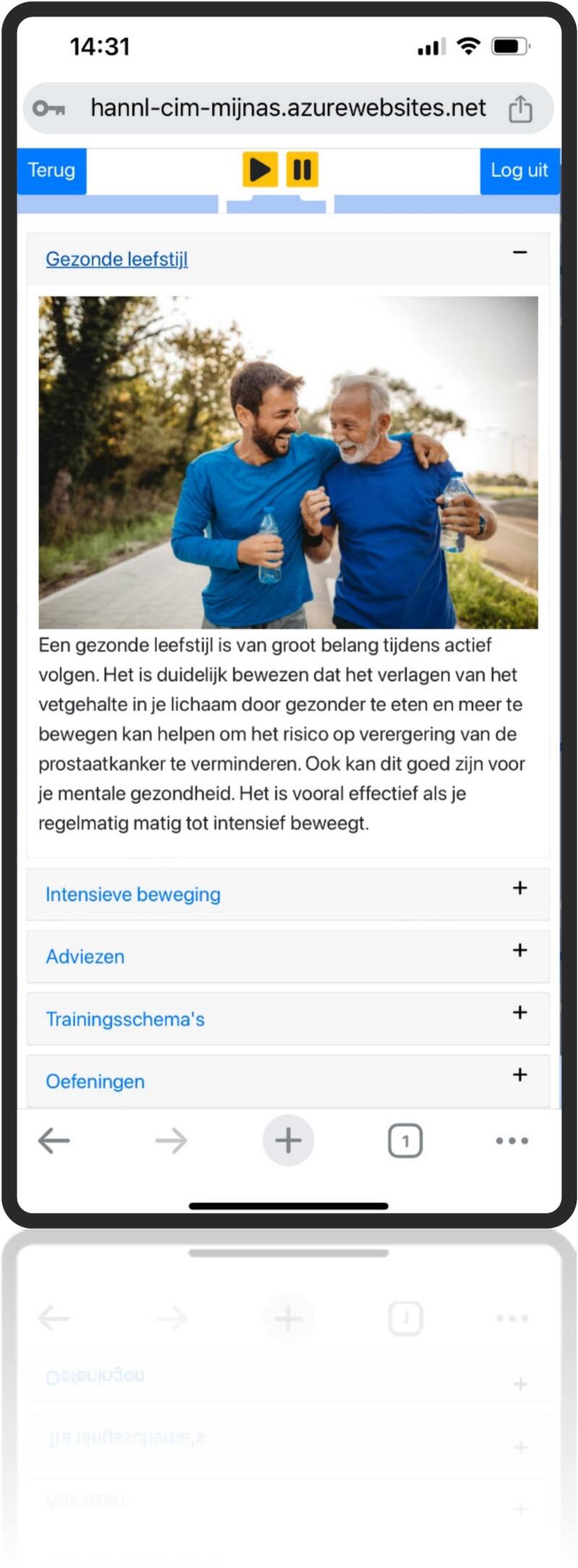
Self management application Lifestyle.

**Fig. 8:**
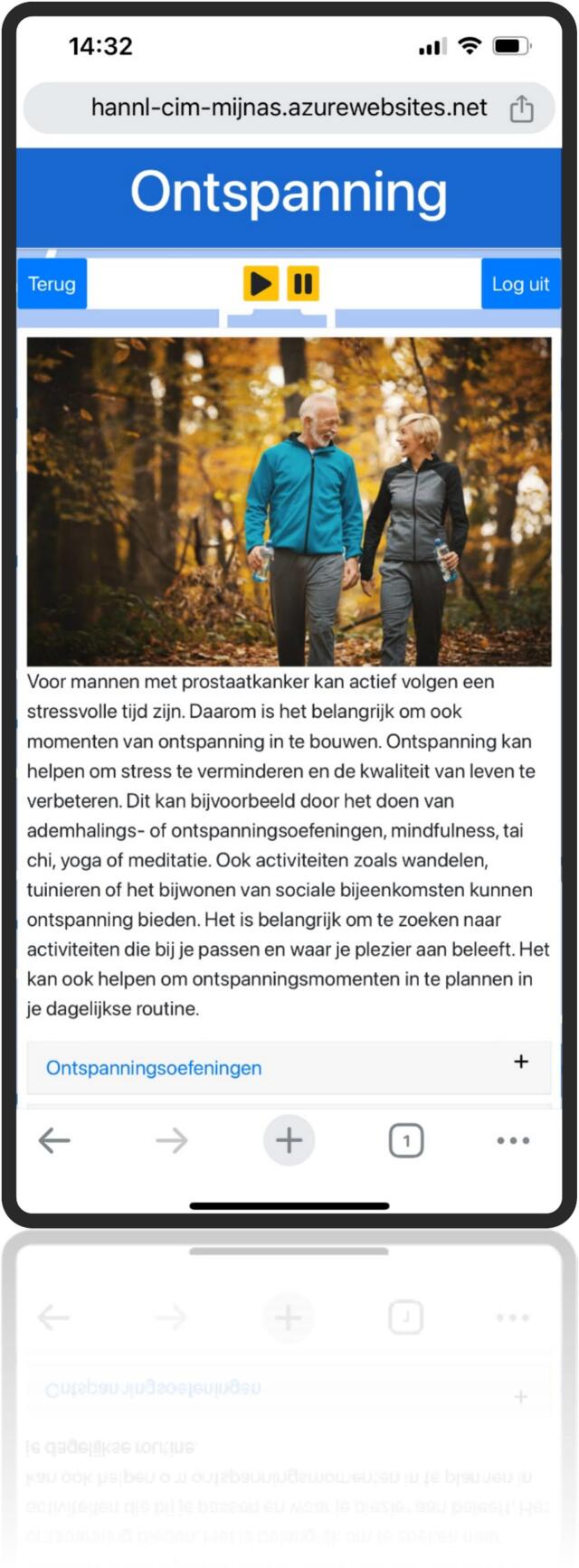
Self management application Relaxation.

**Fig. 9:**
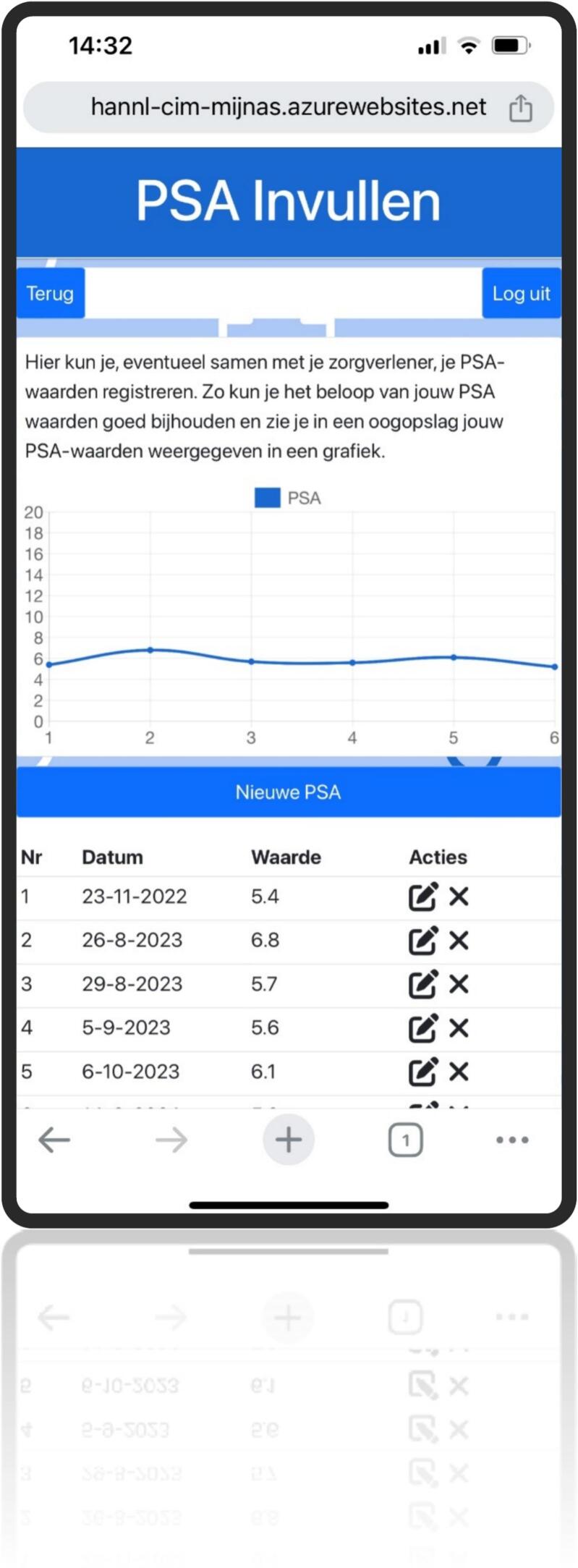
Self management application My PSA test results.

**Fig. 10:**
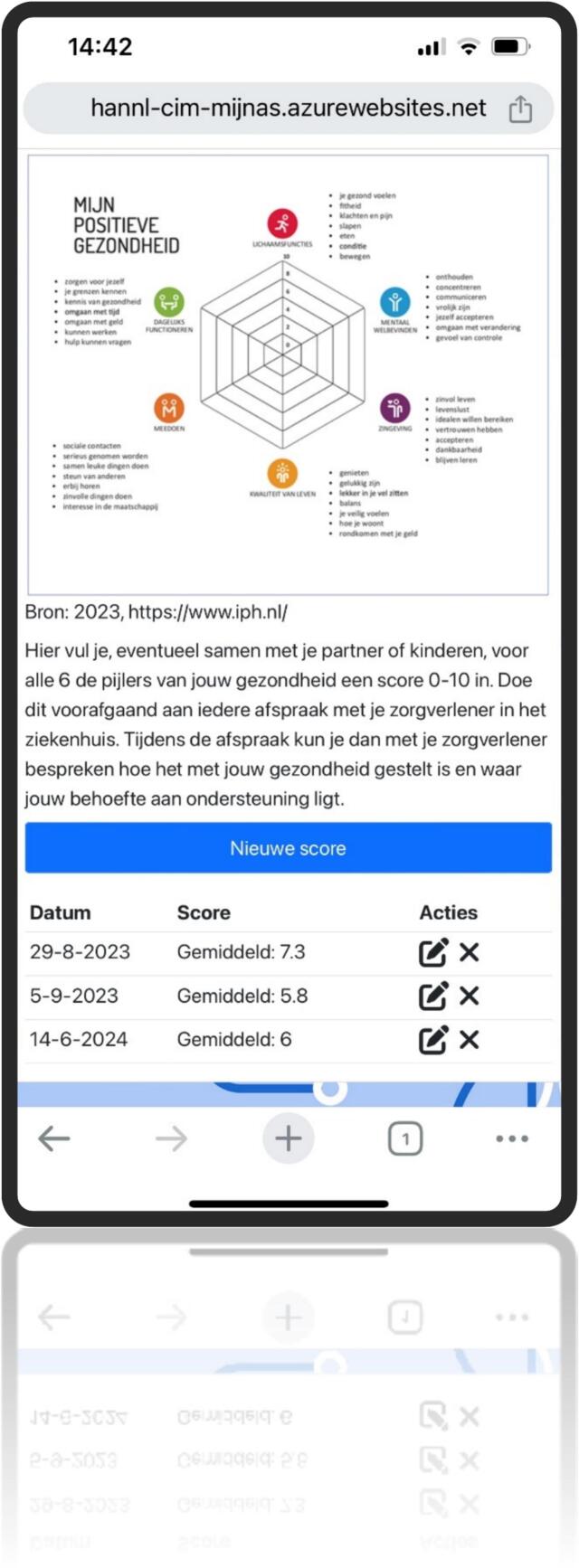
Self management application Prepare my next consultation.

This resulted in the development of a web-based application featuring six primary buttons: information, lifestyle, appointments, medical results, contact information, and personal information. Behind each button, users will find a brief instruction leading to expandable sections. These sections include information, FAQs, animations, images, interactive elements (like a medical results logbook and preparation for caregiver questions and health scores), guidelines, and exercises. Additionally, it includes a text-to-speech feature for all spoken content. This self-management tool enhances personalized care by delivering timely information, encourages positive lifestyle adjustments, and fosters patient empowerment (Fig. 4, Fig. 5, Fig. 6, Fig. 7, Fig. 8, Fig. 9, Fig. 10).

## 4 Discussion

The study aimed to develop a digital psychosocial support program for men with PCa undergoing AS to help them cope with the emotional and social aspects of their diagnosis and treatment journey using DT principles. While standalone interventions exist, a comprehensive, integrated support program is needed to cater to PCa patients' needs and align with the current care landscape that faces challenges such as demographic changes and staff shortages (Donachie et al., 2022a; van der Nat et al., 2024).

The iterative process of DT allowed for continuous feedback and improvement, ensuring the program evolved to meet changing needs (Oliveira et al., 2020). Collaboration with multidisciplinary stakeholders ensured comprehensive consideration of medical, psychological, social, and practical aspects. Creativity and innovation addressed complex challenges that went beyond traditional pathways. The DT process emphasized a human-centered approach, prioritizing understanding of PCa patients' needs and experiences.

The developed self-management application aims to increase personalized care by providing reliable just-in-time information, facilitating positive lifestyle changes, and promoting patient empowerment. The adoption of this e-health application within the current care path may contribute to reducing the psychosocial burden associated with AS (Grapp et al., 2022). Despite these potential benefits, there is some apprehension regarding the uptake of e-health in PCa patients due to their age and digital literacy (Watkins and Xie, 2014). However, previous research already underlined the potential benefit that e-health self-management interventions can provide for this patient population (Kinsella et al., 2018). In addition, the integration of e-health applications in current care introduces numerous potential benefits for individuals undergoing AS. E-health interventions have the capacity to enhance self-efficacy by providing PCa patients with accessible and personalized tools to actively participate in their care (Wilson et al., 2021; Xu et al., 2019). The application's features, such as self-monitoring functionalities and educational resources, contribute to increased engagement by encouraging a sense of empowerment and involvement in the management of their health (van Olmen et al., 2022). It provides patients with information and practical guidelines to facilitate positive lifestyle changes. The application promotes personalized support setting it apart from generic online resources by integrating patient data and feedback into tailored interventions. It offers just-in-time support aligned with emerging needs. For example, providing access to relevant FAQs or visualizations when new PSA results become available. The patient-completed positive health “spider web” further supports consultations, helping healthcare providers identify real-time concerns (e.g., anxiety or physical symptoms) and refer patients to appropriate features such as relaxation or lifestyle modules. Additionally, the application serves as a communication tool, bridging gaps in information exchange and promoting a more informed and collaborative patient-provider relationship (Shaw et al., 2021).

While the user-centered, iterative, and collaborative design approach aligns with contemporary principles of creative problem-solving in healthcare, it is crucial to critically examine both its strengths and limitations. This method can be time-consuming, subjective, and costly. Although DT does not follow a rigid structure or rigid scientific methods, it is important to recognize that its inherent flexibility allows for responsiveness to the complex, dynamic nature of healthcare challenges. This approach allows for the incorporation of diverse perspectives and adapts to user needs through iterative refinement (Voorheis et al., 2022). In addition, it reduces the risk of subjectivity and increases triangulation of data.

Overall, using design thinking to develop a digital psychosocial support program for men with PCa during AS enhances the program's potential to be effective, patient-centered, and sustainable. The clinical usability and acceptability of the self-management application, incorporated in the care path of men with PCa undergoing AS, needs to be determined in a feasibility study prior to the assessment of its effectiveness in a clinical trial.

## 5 Conclusion

This study applied DT principles to develop a complex intervention addressing unmet needs and promoting personalized, patient-centered care. The self-management application aims to empower individuals undergoing AS by providing tailored support for their psychosocial challenges. Successful implementation relies on collaboration between healthcare providers, patients, and technology developers to ensure seamless integration into the existing care pathway. A feasibility study should be conducted to assess the potential usefulness and integration of this application in clinical practice, with insights from the feasibility study incorporated into a clinical trial evaluating the application's effectiveness in reducing psychosocial burden.

## Ethical approval

This study was carried out in accordance with the World Medical Association Declaration of Helsinki (World Medical Association, 2013).

The HAN university of applied sciences ethical committee provided ethical approval for this study (No. ECO 584.09/24).

## Declaration of Generative AI and AI-assisted technologies in the writing process

During the preparation of this work the author(s) used ChatGPT in order to improve readability. After using this tool, the authors reviewed and edited the content as needed and take full responsibility for the content of the published article.

## Funding sources

This work was supported by a research grant from The Dutch Research Council (NWO) under grant number 023.016.048.

## Declaration of competing interest

The authors declare the following financial interests/personal relationships which may be considered as potential competing interests: Mrs. KM Donachie, RN, MSc reports financial support was provided by Dutch Research Council. If there are other authors, they declare that they have no known competing financial interests or personal relationships that could have appeared to influence the work reported in this paper.

## Data Availability

The data related to the present study are available upon reasonable request from the corresponding author (KD).

## Glossary

Active Surveillance: A management strategy for low-risk prostate cancer (LR-PCa) involving regular monitoring of cancer progression through PSA testing, digital rectal exams, MRI imaging, and biopsies. Curative treatment is initiated only upon tumor progression to avoid treatment-related complications.
Prostate-Specific Antigen Test: A protein produced by the prostate gland, often elevated in the blood of men with prostate cancer. PSA testing is used to detect prostate cancer and monitor its progression.
Digital Rectal Exam: A physical examination in which a healthcare provider inserts a finger into the rectum to feel the prostate for abnormalities, such as lumps or hard areas that might indicate prostate cancer.
Prostate Cancer: A type of cancer occurring in the prostate gland, most commonly affecting men. It is categorized into different risk groups (low-risk, intermediate-risk, high-risk) based on tumor characteristics and PSA levels.
Low-Risk Prostate Cancer: A classification of prostate cancer where the tumor is considered to have a low likelihood of spreading or progressing rapidly, often managed through AS rather than immediate treatment.
Biopsy: A medical procedure in which a small sample of prostate tissue is removed for laboratory analysis to confirm the presence of cancer and assess its aggressiveness.
Erectile Dysfunction: The inability to achieve or maintain an erection sufficient for sexual intercourse. It is a potential complication of prostate cancer treatments, particularly surgery or radiation.
Urinary Incontinence: The loss of bladder control, resulting in involuntary leakage of urine. This can occur as a side effect of prostate cancer treatments such as surgery or radiation therapy.
Psycho-Oncological Support: Psychological and social support provided to cancer patients to help them manage the emotional, mental, and social challenges associated with their diagnosis and treatment.
Design Thinking: A human-centered approach used in healthcare for developing patient-centered interventions. It involves five phases: empathy, define, ideate, prototype, and test.
Scoping Review: A type of literature review that maps the key concepts, evidence, and research gaps in a particular area to inform future research or practice.
Delphi Study: A structured communication technique used in research to gather expert opinions through rounds of surveys, allowing for consensus-building on complex topics.
Heuristic Testing: A usability testing method in which experts evaluate a product against predefined usability principles to identify potential issues and areas for improvement.
Self-Management Application: A digital tool designed to help patients manage their health by providing access to information, tracking medical results, facilitating communication with healthcare providers, and promoting healthy lifestyle choices.
Medical Device Regulation: A set of regulatory requirements in the European Union that governs the safety, effectiveness, and quality of medical devices, including software that might be classified as a medical device.
General Data Protection Act: A European Union regulation that sets rules for protecting personal data and privacy, particularly relevant for healthcare applications that handle sensitive patient information.
PSA Values: Measurements of prostate-specific antigen levels in the blood, used to monitor prostate cancer progression or response to treatment.
E-health: The use of digital technologies, such as mobile apps and telemedicine, to improve healthcare delivery, access to health information, and patient engagement in managing their health.
Self-Efficacy: A person's belief in their ability to manage and take control of their health and treatment. High self-efficacy is associated with better adherence to treatment plans and positive health outcomes.
Psycho-Oncological Research: A field of study focused on the psychological and emotional well-being of cancer patients, addressing their mental health and quality of life during and after cancer treatment.
Patient-Centered Care: An approach to healthcare that prioritizes the needs, preferences, and values of the patient, ensuring that treatment is tailored to the individual's overall well-being.
Purposive Sampling: A non-random sampling technique where participants are selected based on specific characteristics relevant to the research question to ensure a diverse and representative sample.
Empathy Map: A tool used in design thinking to synthesize insights from user research by categorizing what users say, think, do, and feel to better understand their experiences and challenges.
SCAMPER Method: A brainstorming technique used to generate creative ideas for improving products or processes by substituting, combining, adapting, modifying, putting to another use, eliminating, or reversing elements.
